# Temporal expression and spatial distribution of the proteoglycan versican during cardiac fibrosis development

**DOI:** 10.1016/j.mbplus.2023.100135

**Published:** 2023-11-10

**Authors:** Athiramol Sasi, Andreas Romaine, Pugazendhi Murugan Erusappan, Arne Olav Melleby, Almira Hasic, Christen Peder Dahl, Kaspar Broch, Vibeke Marie Almaas, Rosa Doñate Puertas, H. Llewelyn Roderick, Ida Gjervold Lunde, Ivar Sjaastad, Maria Vistnes, Geir Christensen

**Affiliations:** a Institute for Experimental Medical Research, Oslo University Hospital and University of Oslo, Oslo, Norway; b K.G. Jebsen Center for Cardiac Research, University of Oslo, Oslo, Norway; c Research Institute of Internal Medicine, Oslo University Hospital, Oslo, Norway; d Department of Cardiology, Oslo University Hospital Rikshospitalet, Oslo, Norway; e Experimental Cardiology, Department of Cardiovascular Sciences, KU Leuven, Leuven, Belgium; f Department of Cardiology, Oslo University Hospital Ullevål, Oslo, Norway; g Center for Clinical Heart Research, Department of Cardiology, Oslo University Hospital Ullevål, Oslo, Norway; h K.G. Jebsen Center for Cardiac Biomarkers, Institute for Clinical Medicine, Campus Ahus, University of Oslo, Oslo, Norway

**Keywords:** Versican, Cardiac fibrosis, Cardiomyopathy, Pressure overload, Extracellular matrix

## Abstract

Cardiac fibrosis is a central pathological feature in several cardiac diseases, but the underlying molecular players are insufficiently understood. The extracellular matrix proteoglycan versican is elevated in heart failure and suggested to be a target for treatment. However, the temporal expression and spatial distribution of versican and the versican cleavage fragment containing the neoepitope DPEAAE in cardiac fibrosis remains to be elucidated. In this study, we have examined versican during cardiac fibrosis development in a murine pressure overload model and in patients with cardiomyopathies. We found that versican, mainly the V1 isoform, was expressed immediately after induction of pressure overload, preceding collagen accumulation, and versican protein levels extended from the perivascular region into the cardiac interstitium. In addition, we found increased production of versican by collagen expressing fibroblasts, and that it was deposited extensively in the fibrotic extracellular matrix during pressure overload. In cardiac cell cultures, the expression of versican was induced by the pro-fibrotic transforming growth factor beta and mechanical stretch. Furthermore, we observed that the proteolytic cleavage of versican (DPEAAE fragment) increased in the late phase of fibrosis development during pressure overload. In patients with hypertrophic and dilated cardiomyopathies, we found elevated levels of versican and a positive correlation between versican and collagen mRNA in the heart, as well as increased cleavage of full-length protein. Taken together, the temporal expression profile and the spatial distribution of both the full-length versican and the DPEAAE fragment observed in this study indicates a role for versican in development of cardiac fibrosis.

## Highlights

• The proteoglycan versican is elevated from the early phase of cardiac remodeling.
• Collagen-producing fibroblasts are the main source of versican expression.
• Versican production extends from the perivascular region into cardiac interstitium.
• The amount of the DPEAAE fragment increases towards the late phase of cardiac remodeling.
• Cardiomyopathy patients have accumulation of versican and DPEAAE in fibrotic areas.

## Graphical abstract

**Figure:**
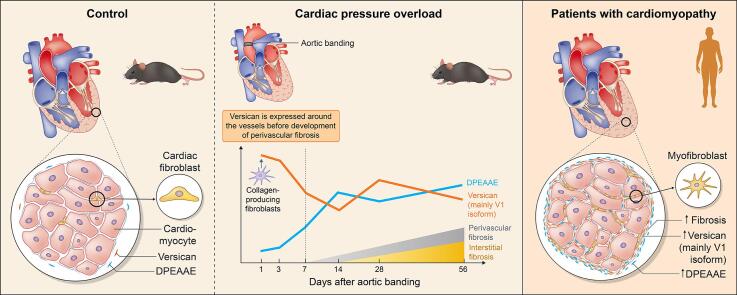
Temporal expression profiles of versican and the DPEAAE fragment during cardiac fibrosis development. The V1 isoform of versican is expressed by collagen-producing fibroblasts early after initiation of pressure overload. Versican is expressed around the vessels before development of perivascular fibrosis. In patients with cardiomyopathies, increased amounts of versican and the DPEAAE fragment were observed in fibrotic regions of the myocardium.

## Introduction

Cardiac fibrosis is a central pathological feature in several cardiac diseases, including heart failure [1]. Extensive extracellular matrix remodeling, as seen in cardiac fibrosis, contributes to stiffening of the myocardium, affecting both diastolic and systolic function. Cardiac fibrosis is observed in conditions following hypertension, myocardial infarction, or aortic valve stenosis as well as in hypertrophic [2] and dilated cardiomyopathies [3]. Moreover, cardiac fibrosis has been shown to be a prognostic marker for patients with cardiac diseases [4], [5]. Currently, there is no specific therapy for cardiac fibrosis in clinical use [6], and better understanding of the molecular players involved in development of cardiac fibrosis is warranted to provide a basis for novel therapies.

Chondroitin sulfate proteoglycans are heavily glycosylated proteins that have been suggested as therapeutic targets in the treatment of heart failure [7], [8]. Versican is one of the main chondroitin sulfate proteoglycans, and it can be present in four different isoforms V0, V1, V2 and V3 [9]. Versican is crucial for cardiac development, and homozygous deletion of versican is embryonically lethal with severe cardiac defects in mice [10], [11]. Previous studies have demonstrated elevated levels of versican in some cardiac diseases [12], [7], and it has been suggested to be involved in liver fibrosis [13]. However, there is limited information about the temporal expression profile and spatial distribution of versican during development of cardiac fibrosis.

Moreover, the versican cleavage product (DPEAAE fragment) generated by ADAMTS (a disintegrin and metalloproteinase with thrombospondin motifs) enzymes may also be involved in development of cardiac fibrosis and heart failure [12], [7]. Our group has shown that inhibition of the ADAMTS4 enzyme reduces cardiac fibrosis and improves cardiac function in a pressure overload model [14]. However, the temporal profile and tissue distribution of the DPEAAE fragment in cardiac fibrosis need to be identified. In this study, we have quantified the temporal profile, spatial distribution, and regulation of both full-length versican and the DPEAAE fragment during development of cardiac fibrosis.

We used a murine cardiac pressure overload model to assess the temporal and spatial distribution of versican expression, isoforms of versican, and the DPEAAE fragment during development of cardiac fibrosis. Moreover, the cellular origin of versican production was identified by fluorescence *in situ* hybridization during early and late phases of cardiac fibrosis development. We also used cardiac cell cultures to determine the potential regulatory factors of versican expression. Finally, we analyzed the expression and spatial distribution of versican and the DPEAAE fragment in myocardial samples from hypertrophic and dilated cardiomyopathy patients.

## Results

### **Cardiac fibrosis development following pressure overload and spatio-temporal distribution of versican**

#### Pressure overload induces fibrosis and reduces cardiac function

**Fig. 1:**
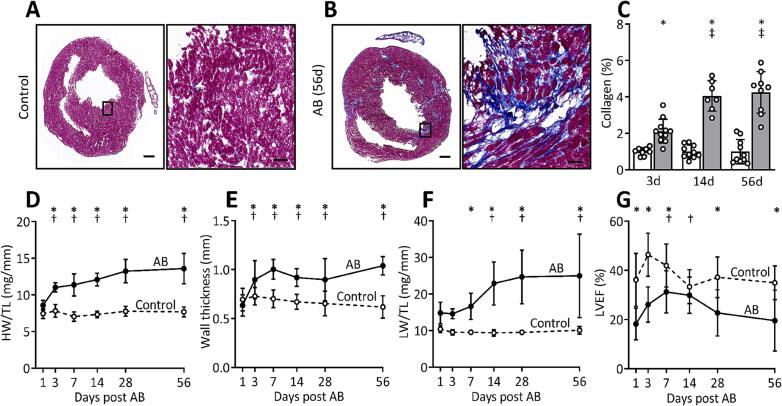
Pressure overload induces fibrosis and cardiac dysfunction. (A, B) Representative Masson’s trichrome stained images of hearts 56 days after (A) sham operation (Control) or (B) aortic banding (AB). Scale bar = 500 µm, 50 µm (insets). (C) Bar graph shows quantification of collagen (calculated as percentage of collagen positive area divided by total area) from Control (white bar, n = 8–10) and AB (grey bar, n = 7–11) hearts at day 3, 14 and 56 after AB, presented as values relative to respective Control. (D) Heart weight (HW) normalized to tibia length (TL), (E) left ventricular posterior wall thickness in diastole (Wall thickness), (F) lung weight (LW) normalized to TL, (G) left ventricular ejection fraction (LVEF) in Control (n = 9–10) and AB (n = 8–11) hearts. Echocardiographic measurements were obtained from Control (n = 18–20 at day 1 to 28, n = 10 at day 56) and AB hearts (n = 16–18 at day 1 to 28, n = 8 at day 56). Data represent mean ± SD. Repeated measures two-way ANOVA with Bonferroni’s multiple comparisons test was used for statistical analysis. P values < 0.05 were considered statistically significant. *P < 0.05 Control vs. AB, ^†^P < 0.05 AB vs. day 1 post AB, ^‡^P < 0.05 AB vs. day 3 post AB.

To assess cardiac fibrosis development during pressure overload, we analyzed mouse hearts at different time points after aortic banding. Cardiac cryosections stained with Masson’s trichrome showed an increased deposition of collagen in aortic banded hearts compared to control mice (Fig. 1A, B). The accumulation of collagen increased from day 3, doubled at day 14, and remained at the same level until day 56 after aortic banding (Fig. 1C). The aortic banded mice also had a progressive increase in heart weight and left ventricular wall thickness from the third day until end of study at day 56 (Fig. 1D, E). Moreover, increasing lung weight (Fig. 1F), larger left atrial diameter (Fig. S1A), as well as reduced left ventricular ejection fraction (Fig. 1G) and fractional shortening (Fig. S1B) indicated development of cardiac dysfunction and heart failure. Upregulation of cardiac remodeling markers in aortic banded mice are shown in Fig. S1C-F.

#### Versican mRNA expression is increased immediately after induction of pressure overload

**Fig. 2:**
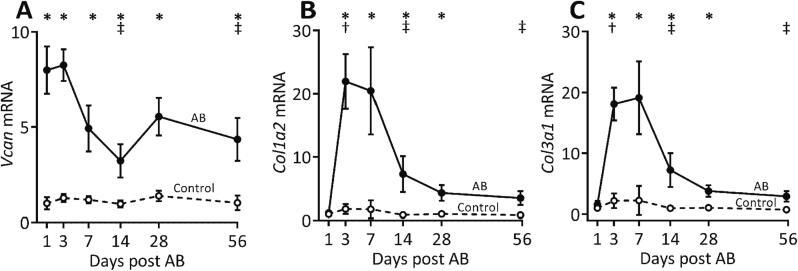
Versican expression is increased and precedes collagen expression in the early phase of pressure overload. (A) mRNA expression of versican (*Vcan*, measured using primer pairs targeting all isoforms of *Vcan*), (B) collagen I alpha 2 (*Col1a2*), and (C) collagen III alpha 1 (*Col3a1*) in the left ventricles of mouse hearts at day 1, 3, 7, 14, 28 and 56 after aortic banding (AB, n = 8–11) or sham operations (Control, n = 9–10). The absolute quantity of gene expression (copies/μl measured by ddPCR) relative to respective Control at day 1 is presented. Data represent mean ± SD. Repeated measures two-way ANOVA with Bonferroni’s multiple comparisons test was used for statistical analysis. P values < 0.05 were considered statistically significant. *P < 0.05 Control vs. AB, ^†^P < 0.05 AB vs. day 1 post AB, ^‡^P < 0.05 AB vs. day 3 post AB.

We found an 8-fold higher versican expression at day 1 after aortic banding than in control hearts. Even though the mRNA level of versican remained high at every time point in the aortic banded hearts, the expression was reduced at day 14 and 56 compared to day 3 after aortic banding (Fig. 2A). Furthermore, the mRNA expression of collagen types I and III increased 12-fold (Fig. 2B) and 8-fold (Fig. 2C), respectively, at day 3. This increase in collagen expression occurred after the initial increase in versican. The mRNA expression of versican correlated with collagen I and collagen III, respectively, from day 3 to 14 after aortic banding (Fig. S2A-B, D-E), but not in the late phase of fibrosis development (day 56) (Fig. S2C, F).

#### V0 and V1 are the main isoforms of versican expressed during cardiac fibrosis development

**Fig. 3:**
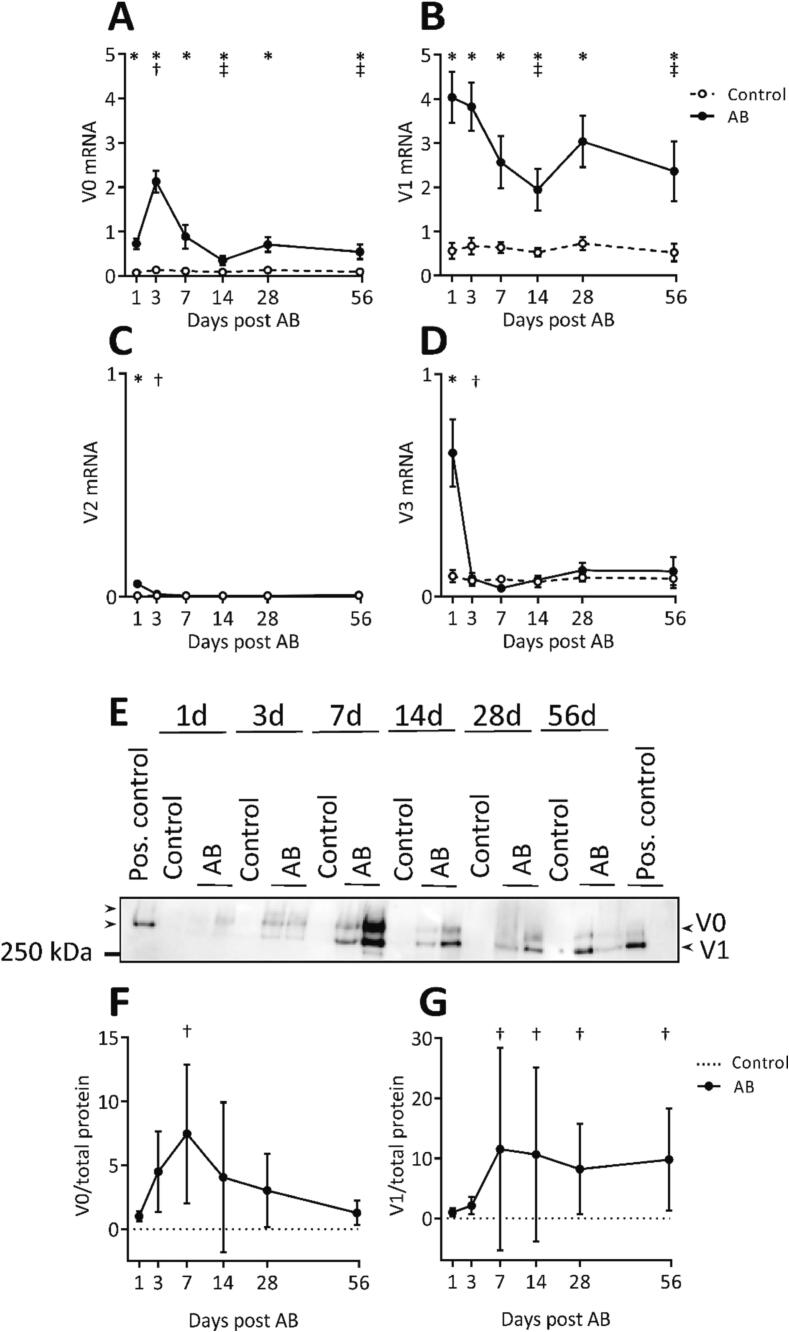
V0 and V1 are the main isoforms expressed during fibrosis development during pressure overload. (A-D) mRNA expression of versican isoforms V0, V1, V2 and V3 from the left ventricle of mouse hearts at day 1, 3, 7, 14, 28 and 56 after aortic banding (AB, n = 8–11) or sham operations (Control, n = 9–10). (E-G) Representative western blot image and quantification (normalized to total protein stain, relative to day 1 post AB) of the V0 and V1 isoform from the left ventricles of mice at day 1, 3, 7, 14, 28 and 56 after aortic banding (AB, n = 8) or sham operations (Control, n = 5–8). All samples were treated with chondroitinase ABC enzyme to remove glycosaminoglycan chains, except for the sample on the right side of the image. Pooled extracellular matrix fractions from left ventricular tissue of aortic banded mice obtained after 8 to 10 weeks were used as positive controls. Control samples (sham operation) displayed very low or no amount of versican. The absolute quantity of mRNA expression (copies/μl measured by ddPCR) relative to mRNA expression measured by primer pairs targeting all isoforms of *Vcan* is presented. Data represent mean ± SD. Repeated measures two-way ANOVA (A-D) or one-way ANOVA (F) with Bonferroni’s multiple comparisons test, and Kruskal-Wallis (G) with Dunn’s post-hoc test were used for statistical analysis. P values < 0.05 were considered statistically significant. *P < 0.05 Control vs. AB, ^†^P < 0.05 AB vs. day 1 post AB, ^‡^P < 0.05 AB vs. day 3 post AB. Note the differences in the scaling of y-axis of the line graphs (A-D and F-G).

We found that the V0 and V1 versican isoforms were substantially upregulated during cardiac pressure overload. The mRNA expression of the V1 (7.2-fold increase) and the V0 (16-fold increase) isoforms peaked at day 1 and 3 after aortic banding, respectively. After the initial increase, both isoforms remained elevated from day 7 to 56 in the aortic banded hearts compared to control mice (Fig. 3A, B). The expression of V2 and V3 isoforms were much lower than the expression of V0 and V1 isoforms (Fig. 3C, D). The protein amount of the V0 isoform peaked at day 7 and gradually declined from day 14 to 56 in the aortic banded hearts (Fig. 3E-G). The V1 isoform increased at day 7 and remained high after aortic banding.

#### Collagen expressing fibroblasts are the main producers of versican in the initial and late phase of cardiac fibrosis

**Fig. 4:**
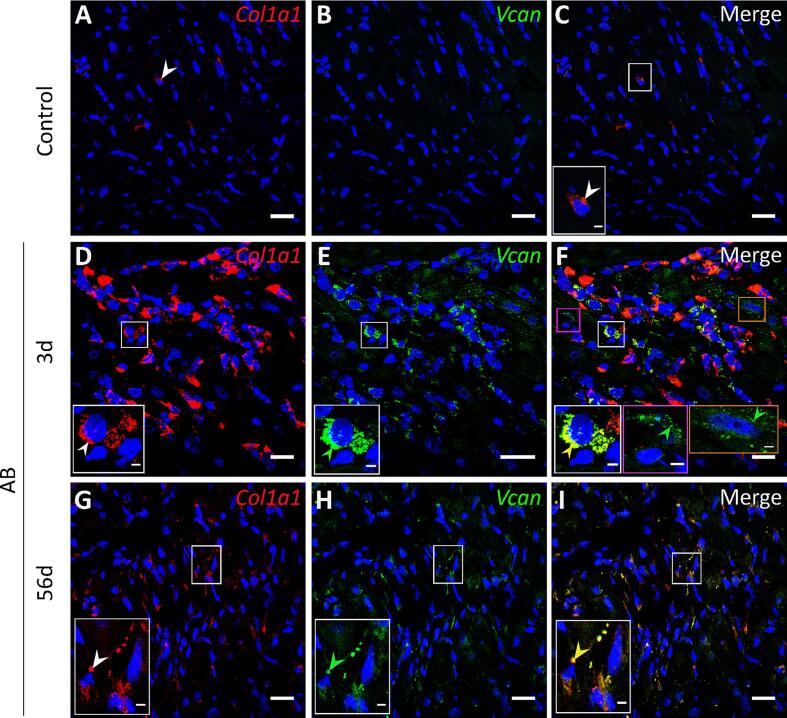
Cellular origin of versican during development of cardiac fibrosis determined by single molecular fluorescence *in situ* hybridization (smFISH). Representative confocal images of *Col1a1* (red), and *Vcan* (green) in the heart after sham operation (A-C, Control, n = 3) and aortic banding at day 3 (D-F, AB, n = 3) and at day 56 (G-I). DAPI stain for the nuclei (blue). (F) Versican expression by non-cardiomyocytes and cardiomyocytes after 3 days of aortic banding is shown in pink and orange boxes, respectively. The white arrows show positive signal for *Col1a1* (A, D, G), green arrows show positive signal for *Vcan* (E, H), and yellow arrows show colocalization of *Col1a1* and *Vcan* (F, I). Scale bar = 20 μm, 3 μm (insets). (For interpretation of the references to color in this figure legend, the reader is referred to the web version of this article.)

We examined the cellular origin of versican in the early and late phase of cardiac remodeling by single molecular fluorescence *in situ* hybridization (smFISH) using probes targeting *Vcan* and *Col1a1* (a fibroblast marker) [15]. In the early phase of cardiac remodeling (day 3 after aortic banding), FISH probes targeting the *Vcan* gene were detected in multiple cell types including fibroblasts (Fig. 4F, shown in white box, identified based on *Col1a1* expression in 4D), cardiomyocytes (Fig. 4F, shown in orange box, identified based on nuclear size by DAPI staining and distance from neighboring cell nucleus), and non-cardiomyocytes (Fig. 4F, shown in pink box, identified based on the nuclear size and negative for *Col1a1*) in the fibrotic regions compared to control hearts which showed modest expression of collagen and versican (Fig. 4A-C). We observed that the majority of the cells expressing *Vcan* co-express *Col1a1* (Fig. 4D, E) indicating that versican is synthesized mostly by fibroblasts. In the late phase (day 56 after aortic banding), versican expression was mainly observed in *Col1a1* expressing cells in the fibrotic areas (Fig. 4G-I). FISH probes targeting GFP mRNA (Fig. S3A-C) were used as negative controls.

#### TGF-β1 and mechanical stretch induce versican expression in cardiac cells

**Fig. 5:**
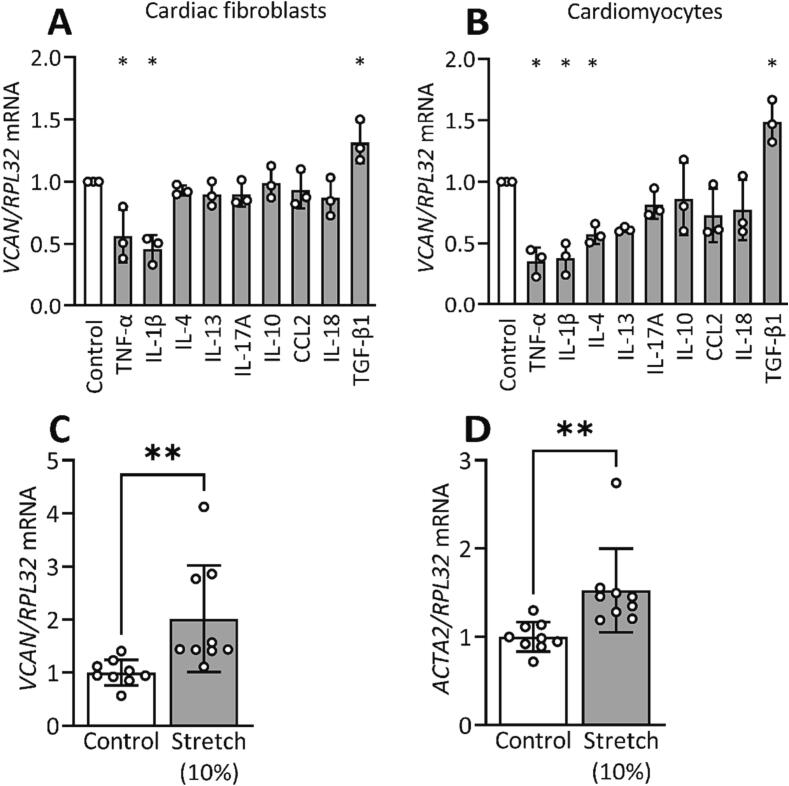
Versican (*VCAN*) expression is regulated by cytokines and mechanical stretch in cardiac cells. (A) mRNA expression of versican was upregulated by TGF-β1, and downregulated by TNF-α, and IL-1β in cardiac fibroblasts, and (B) in addition by IL-4 in cardiomyocytes compared to control (Control, n = 3). (C, D) Versican and α-SMA (*ACTA2*) mRNA expression were increased after biaxial strain of 10% (stretch) for 24 h at 1 Hz frequency in fibroblasts compared to non-stretched cells (Control, n = 9). Gene expression is presented as values relative to Control. Bar graphs represent mean ± SD. One-way ANOVA with Dunnett’s multiple comparisons test (A, B) and Student’s *t*-test (C, D) were used for statistical analysis. P values < 0.05 were considered statistically significant. *P < 0.05 Control vs. cytokine treatment or stretch.

Since mechanical stress and inflammation are central factors involved in development of cardiac fibrosis [16], [17], we determined whether versican expression could be induced by these factors. We found that fibroblasts and cardiomyocytes have higher versican expression at day 3 after aortic banding. The levels of inflammatory cell infiltration have been shown to be increased following aortic banding [18], [19], [20] and mechanical stress of cardiac cells is evident. Thus, to examine the regulation of versican expression at the cellular level, we treated human cardiac fibroblasts and cardiomyocytes with a panel of inflammatory cytokines and growth factors that belong to Th1/Th2/Th17/Treg mediated immune profile (Fig. 5A, B). We found that TNF-α and IL-1β reduced versican expression, whereas TGF-β1 increased the expression substantially in fibroblasts and cardiomyocytes (Fig. 5A, B). Moreover, IL-4 downregulated versican expression in cardiomyocytes (Fig. 5B). Next, we investigated the effect of mechanical stress on versican expression using human cardiac fibroblasts. Our data showed that biaxial stretch induced versican and α-SMA expression, a biomarker of fibroblast activation [21], in human cardiac fibroblasts (Fig. 5C, D).

#### Versican extends from the perivascular region during fibrosis development

**Fig. 6:**
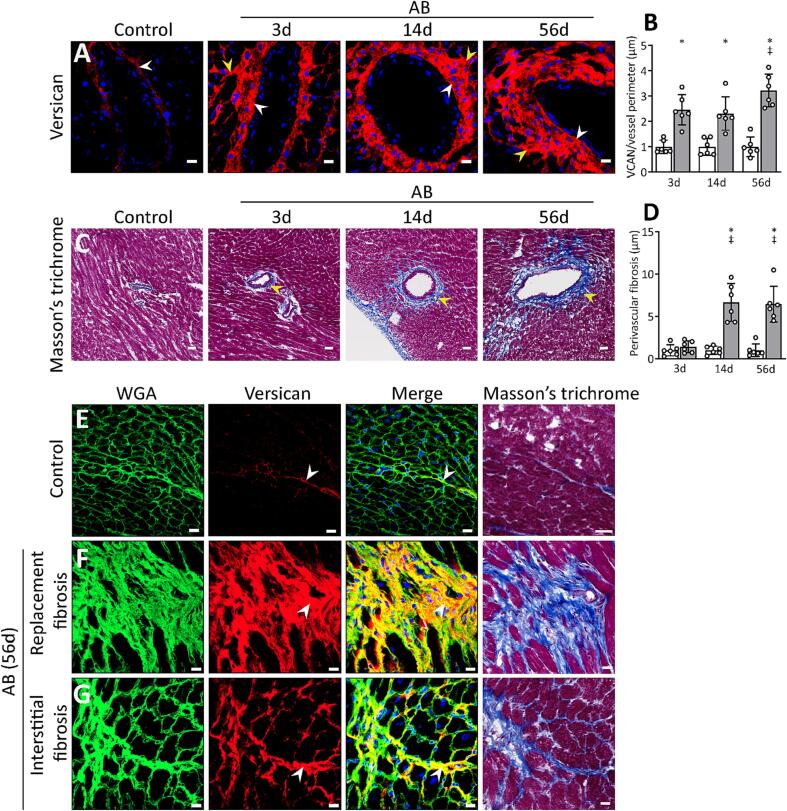
Versican (VCAN) accumulates in perivascular regions, and in areas of replacement and interstitial fibrosis in aortic banded hearts. (A, B) Representative confocal images of versican (red), and quantification (normalized to perimeter of the vessel, and relative to respective Control) in the cardiac perivascular region of mice after sham (Control, white bar, n = 6) and aortic banding operations (AB, grey bar, n = 6) at day 3, 14 and 56. The white arrows show staining for versican in the tunica adventitia of the vessels, yellow arrows show versican in the surrounding interstitium. (C, D) Representative Masson’s trichrome stained images showing perivascular fibrosis, and quantification (fibrotic area normalized to perimeter of vessel, and relative to respective Control) of Control (white bar, n = 6) and AB hearts (grey bar, n = 6) at day 3, 14 and 56. The yellow arrows show progression of perivascular fibrosis into the surrounding interstitium after aortic banding. Scale bar = 35 μm. (E-G) Representative confocal images of versican and Masson’s trichrome stained images from Control and AB hearts after 56 days showing (F) replacement and (G) interstitial fibrosis. Wheat germ agglutinin (WGA, green) stain for the cardiomyocyte sarcolemma and extracellular matrix, versican (red), and DAPI stain for the nuclei (blue). The white arrows show positive staining for versican and yellow color shows colocalization at fibrotic regions. Scale bar = 15 μm for confocal images, 20 μm for Masson’s trichrome stained images. Data represent mean ± SD. Repeated measures two-way ANOVA with Bonferroni’s multiple comparisons test was used for statistical analysis. P values < 0.05 were considered statistically significant. *P < 0.05 Control vs. AB, ^‡^P < 0.05 AB vs. day 3 post AB. (For interpretation of the references to color in this figure legend, the reader is referred to the web version of this article.)

We next examined the spatial distribution of versican in cardiac fibrosis by immunostaining of aortic banded hearts. Versican protein levels were already increased in the perivascular region at day 3 in the aortic banded hearts (Fig. 6A, B). At day 56, we observed higher levels of versican around the cardiac vessels than at day 14, suggesting that versican extends from the perivascular region to the surrounding interstitium. Perivascular fibrosis was more pronounced from day 14 to 56 after aortic banding, than at day 3 in aortic banded hearts (Fig. 6C, D). The immunostained mouse cardiac cryosections obtained at day 56 showed an excessive accumulation of versican both in areas of replacement (accumulation of fibrous tissue to replace cardiomyocyte death) and interstitial fibrosis (accumulation of fibrotic extracellular matrix between the cardiomyocytes) (Fig. 6F, G), whereas the amount of versican was low in control hearts (Fig. 6E).

#### Versican in hypertrophic obstructive cardiomyopathy (HOCM)

**Fig. 7:**
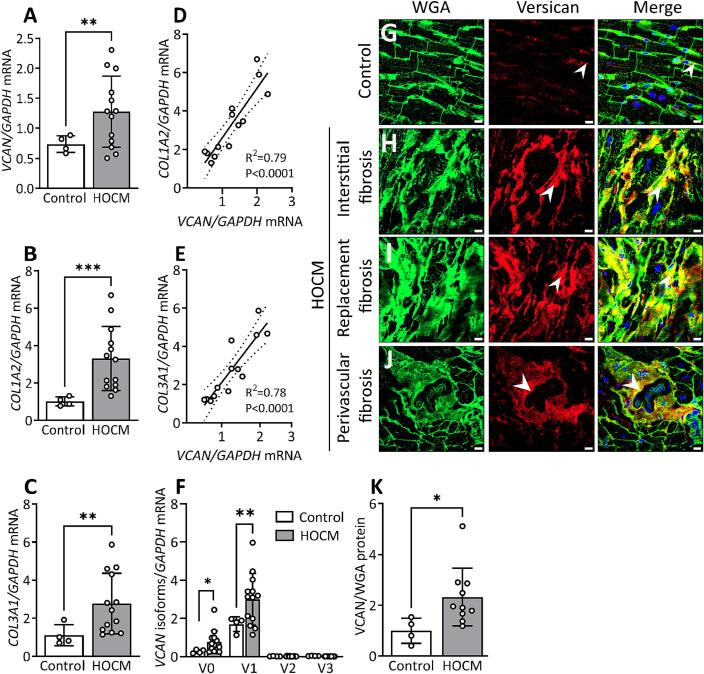
Production and localization of versican (*VCAN*) in patients with hypertrophic obstructive cardiomyopathy (HOCM). (A) Relative mRNA expression of *VCAN*, (B) collagen I alpha 2 (*COL1A2*), (C) and collagen III alpha 1 (*COL3A1*) in the left ventricular tissue from patients with HOCM (n = 13) compared to control hearts (Control, n = 4). (D) Correlation (R^2^) between relative mRNA expression of *VCAN* and *COL1A2*, and (E) *COL3A1* in HOCM. (F) mRNA expression of versican isoforms V0, V1, V2, V3 in HOCM (n = 13) and Control (n = 4). The values are relative to average of total versican expression in control hearts. (G) Representative confocal images of versican (red) from Control left ventricle tissue and HOCM with (H) interstitial, (I) replacement and (J) perivascular fibrosis. (K) Quantification of versican protein level in HOCM (n = 10) versus Control (n = 4) from the confocal images. Positively stained area for versican was normalized to wheat germ agglutinin (WGA, green) stain for cardiomyocyte sarcolemma and extracellular matrix. Values relative to the average of Control are reported. Data points in the bar graph (K) represent the average value from n = 8 images per sample. DAPI stain for the nuclei (blue). White arrows show positive staining for versican and yellow color shows colocalization at fibrotic regions. Scale bar = 15 μm. Bar graph with scatter plot shows mean ± SD. Welch’s *t*-test (A, B, C, F), Student’s *t*-test (K), and Pearson correlation coefficient (D, E) were used for statistical analysis. P values < 0.05 were considered statistically significant. *P < 0.05, **P < 0.01, ***P < 0.001 Control vs. HOCM. (For interpretation of the references to color in this figure legend, the reader is referred to the web version of this article.)

To assess the translational relevance of our findings, we examined the production and localization of versican in cardiac tissue from patients with cardiomyopathies. Molecular analysis of myocardial samples from patients with HOCM showed a 1.7-fold upregulation of total versican (Fig. 7A), a 3.2-fold upregulation of collagen I (Fig. 7B) and 2.5-fold increase of collagen III at the mRNA level (Fig. 7C). Moreover, the mRNA expression of versican correlated positively with mRNA expression of collagen I (Fig. 7D) and collagen III (Fig. 7E) in these patients. Next, we investigated the expression of versican isoforms in patients with HOCM. We found an increase of V0 (2.3-fold) and V1 (1.7-fold) isoforms in the myocardium of patients with HOCM compared to control hearts (Fig. 7F). The expression of V2 and V3 isoforms was found to be low in control hearts with no increase in patients with HOCM.

Low levels of versican protein were observed in the myocardium of control hearts (Fig. 7G). In patients with HOCM, versican was localized to the fibrotic areas, including, interstitial (Fig. 7H), replacement (Fig. 7I) and perivascular fibrotic regions (Fig. 7J). Furthermore, we observed accumulation of versican in tunica adventitia of the arteries (Fig. 7J). Quantification of immunostained cardiac cryosections showed a 2.3-fold upregulation of versican protein levels (Fig. 7K) in patients with HOCM compared to control hearts.

#### Versican in dilated cardiomyopathy (DCM)

**Fig. 8:**
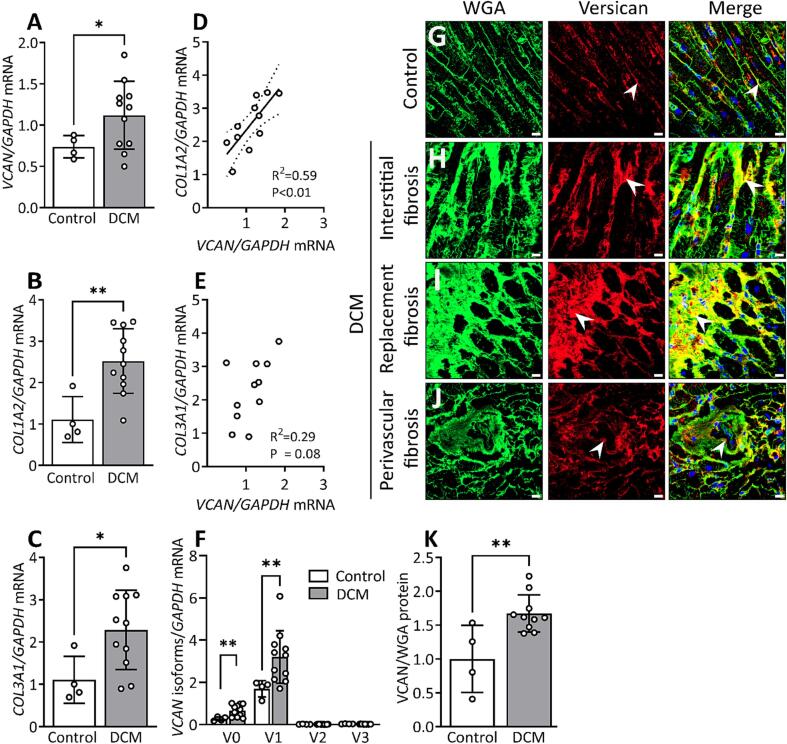
Expression and localization of versican (*VCAN*) in patients with dilated cardiomyopathy (DCM). (A) Relative mRNA expression of *VCAN*, (B) collagen I alpha 2 (*COL1A2*), (C) collagen III alpha 1 (*COL3A1*) in left ventricular tissue from patients with DCM (n = 11) compared to control hearts (Control, n = 4). (D) Correlation (R^2^) between relative mRNA expression of *VCAN* and *COL1A2*, and (E) *COL3A1* in DCM. (F) mRNA expression of versican isoforms V0, V1, V2, V3 in DCM (n = 11) and Control (n = 4). The values are relative to average of total versican expression in control hearts. (G) Representative confocal images of versican (red) from Control left ventricle tissue, and DCM with (H) interstitial, (I) replacement and (J) perivascular fibrosis. (K) Quantification of versican protein level in DCM (n = 10) versus Control (n = 4) from the confocal images. Positively stained area for versican was normalized to wheat germ agglutinin (WGA, green) stain for cardiomyocyte sarcolemma and extracellular matrix. Values relative to the average of Control are reported. Data points in the bar graph (K) represent the average value from n = 8 images per sample. DAPI stain for the nuclei (blue). White arrows show positive staining for versican and yellow color shows colocalization at fibrotic regions. Scale bar = 15 μm. Bar graph with scatter plot shows mean ± SD. Welch’s *t*-test (A, B, C, F), Student’s *t*-test (K), and Pearson correlation coefficient (D, E) were used for statistical analysis. P values < 0.05 were considered statistically significant. *P < 0.05, **P < 0.01 Control vs. DCM. (For interpretation of the references to color in this figure legend, the reader is referred to the web version of this article.)

In patients with dilated cardiomyopathy, we found a 1.5-fold increase in versican mRNA expression (Fig. 8A), a 2.27-fold increase of collagen I mRNA (Fig. 8B), and a 2-fold upregulation of collagen III mRNA (Fig. 8C) compared to control hearts. Moreover, the mRNA expression of versican correlated positively with mRNA expression of collagen I (Fig. 8D), but not with collagen III (Fig. 8E) in patients with DCM. The upregulation of versican was primarily found for the V0 and V1 isoforms, with a 2-fold increase in the mRNA expression of the V0 isoform and a 1.8-fold increase in the V1 isoform (Fig. 8F). As opposed to HOCM with myocardial disarray [22], [23], we observed that cardiomyocytes were aligned in patients with DCM (Fig. 8H). Furthermore, we found increased deposition of ECM within areas of replacement, interstitial and perivascular fibrosis in DCM compared to control hearts. The abundance of versican protein was increased by 1.6-fold (Fig. 8K) and was localized to interstitial, replacement, and perivascular fibrotic regions (Fig. 8H-J) in patients with DCM compared to control hearts (Fig. 8G).

## Production and localization of the versican cleavage fragment (DPEAAE) during cardiac fibrosis development in mice and in patients with cardiomyopathies

### *DPEAAE is upregulated and accumulated in the late phase of fibrosis development in mice following aortic banding*

**Fig. 9:**
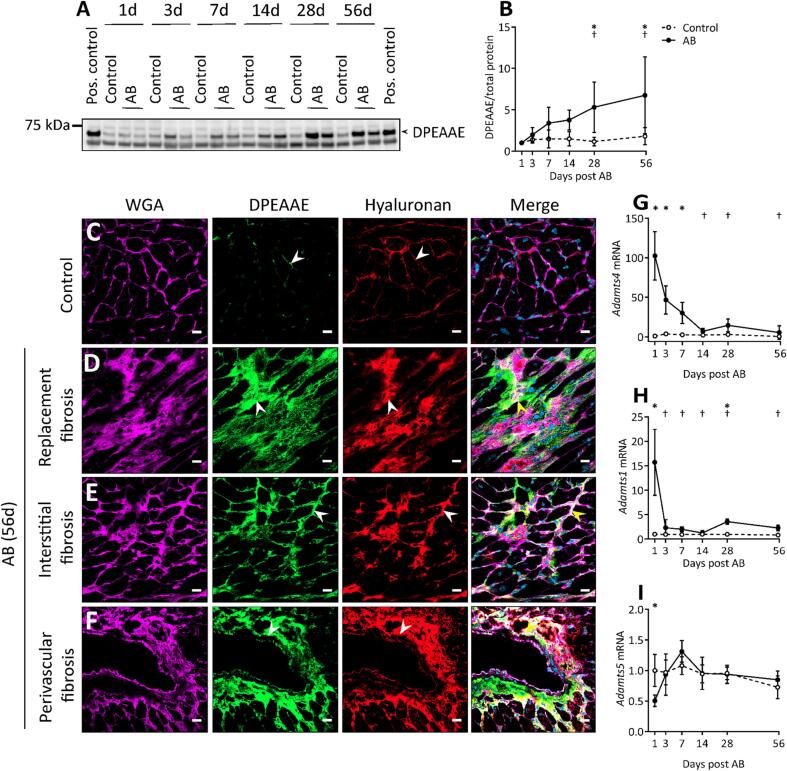
DPEAAE in the fibrotic extracellular matrix of aortic banded hearts. (A, B) Representative western blot image and quantification (normalized to total protein stain, values were relative to day 1 post AB and sham operations, respectively) of DPEAAE in the 1% SDS fraction of left ventricular tissue lysate from sham operation (Control, n = 5–8) and aortic banding (AB, n = 8) at day 1, 3, 7, 14, 28 and 56. (C) Representative confocal images of DPEAAE and hyaluronan from Control and aortic banded hearts after 56 days with (D) replacement, (E) interstitial, and (F) perivascular fibrosis. Wheat germ agglutinin (WGA, magenta) stain for the cardiomyocyte sarcolemma and extracellular matrix, DPEAAE (green), hyaluronan (red), and DAPI stain for the nuclei (blue). White arrows show deposition of DPEAAE and hyaluronan in the extracellular matrix and yellow arrows show colocalization at fibrotic regions. Scale bar = 15 μm. Relative mRNA expression of *Adamts4* (G), *Adamts1* (H), and *Adamts5* (I) from the left ventricle of mouse hearts at day 1, 3, 7, 14, 28 and 56 after aortic banding (AB, n = 8–11) or sham operations (Control, n = 9–10). The values (copies/µl measured by ddPCR) are relative to the respective Control hearts at day 1. Data represent mean ± SD. Repeated measures two-way ANOVA with Bonferroni’s multiple comparisons test was used for statistical analysis. P values < 0.05 were considered statistically significant. *P < 0.05 Control vs. AB, ^†^P < 0.05 AB vs. day 1 post AB. (For interpretation of the references to color in this figure legend, the reader is referred to the web version of this article.)

Since the cleavage product of versican is thought to have a functional role [24], [25], we next sought to determine the spatial and temporal accumulation of DPEAAE fragment during fibrosis development. We found a gradual increase in DPEAAE protein levels from day 7 to 56 after aortic banding compared to control mice. At day 56 after aortic banding, DPEAAE showed a 6-fold increase compared to day 1 after aortic banding (Fig. 9A, B). Immunostaining showed that, similar to full-length protein, DPEAAE was increased and accumulated in replacement, interstitial and perivascular fibrotic regions (Fig. 9D-F), whereas the amount of the DPEAAE fragment was low in control mice (Fig. 9C). In contrast to full-length protein, DPEAAE was found specifically in tunica intima and adventitia of cardiac vessels during development of perivascular fibrosis (Fig. 9F). Moreover, we found that accumulation of hyaluronan was increased and colocalized with DPEAAE after aortic banding (Fig. 9D-F).

Since the DPEAAE fragment derives from cleavage by ADAMTS1, ADAMTS4, and ADAMTS5 [26], we measured the temporal expression of these enzymes in control mice and in aortic banded mice. We found that *Adamts4* displayed the highest expression compared to *Adamts1* and *Adamts5* in the hearts after aortic banding. The expression of the *Adamts4* peaked at day 1 and remained high at day 3 and day 7 after aortic banding compared to control mice (Fig. 9G). The mRNA expression of *Adamts4* then decreased from day 14 to 56 in the aortic banded hearts (Fig. 9G). Furthermore, we observed a higher *Adamts1* expression (Fig. 9H), but a lower *Adamts5* expression at day 1 in the aortic banded hearts compared to control mice (Fig. 9I). There was no significant difference in the mRNA expression of *Adamts1* and *Adamts5* from day 3 to 56 in the aortic banded hearts, except for a slight increase in *Adamts1* expression at day 28 (Fig. 9H, I).

### *DPEAAE in hypertrophic and dilated cardiomyopathy patients (HOCM and DCM)*

**Fig. 10:**
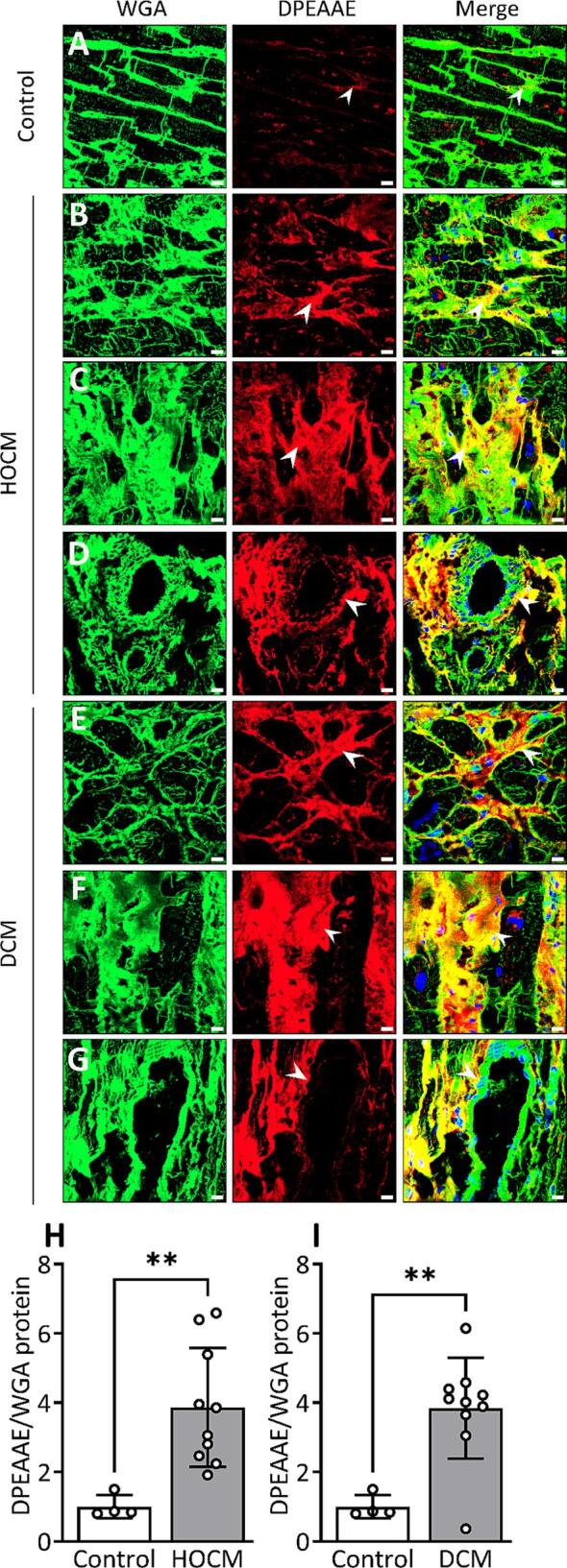
Production and localization of DPEAAE in hypertrophic obstructive and dilated cardiomyopathy patients (HOCM, DCM). (A) Representative confocal images of DPEAAE (red) from control left ventricle tissue (Control) and HOCM with (B) interstitial, (C) replacement and (D) perivascular fibrosis. (E-G) Representative confocal images of DPEAAE (red) from left ventricular tissue of DCM with (E) interstitial, (F) replacement and (G) perivascular fibrosis. (H) Quantification of DPEAAE protein level in HOCM (n = 10) and (I) DCM (n = 10) versus Control (n = 4) from the confocal images. Positively stained area for DPEAAE was normalized to wheat germ agglutinin (WGA, green) stain for cardiomyocyte sarcolemma and extracellular matrix. Values relative to the average of Control are reported. Data points in the bar graph (H, I) represent the average value from n = 8 images per sample. DAPI stain for the nuclei (blue). White arrows show positive staining for DPEAAE and yellow color shows colocalization at fibrotic regions. Scale bar = 15 μm. Bar graph with scatter plot shows mean ± SD. Student’s *t*-test (H, I) was used for statistical analysis. P values < 0.05 were considered statistically significant. **P < 0.01 Control vs. HOCM or Control vs. DCM. (For interpretation of the references to color in this figure legend, the reader is referred to the web version of this article.)

Since the cleavage of versican was prominent towards the late phase of fibrosis development in pressure overload, we also examined DPEAAE in cardiomyopathy patients with established fibrosis. Quantification of immunostained myocardial sections showed a 4.4-fold (Fig. 10H) and 3.8-fold (Fig. 10I) increase of the fragment in patients with HOCM and DCM, respectively. The increase was most prominent in fibrotic regions with interstitial fibrosis and regions of replacement and perivascular fibrosis (Fig. 10B-D in HOCM and Fig. 10E-G in DCM respectively). Moreover, the fragment was found in the tunica adventitia and intima of cardiac vessels in areas with perivascular fibrosis (Fig. 10D and G).

## Discussion

In this study, we have examined the spatial distribution and temporal expression of versican during cardiac fibrosis development following pressure overload in mice and in patients with cardiomyopathies. The main findings of this study are: (1) versican, in particular the V1 isoform, is substantially elevated during the early phase after the induction of cardiac pressure overload, (2) collagen-producing fibroblasts are the main cells expressing versican in cardiac fibrosis, (3) versican synthesis is induced by TGF-β1 and mechanical stretch of cardiac cells, (4) versican extends from the perivascular region to the surrounding interstitium during fibrosis development, (5) the ADAMTS-derived versican cleavage fragment containing the neoepitope DPEAAE is elevated and accumulated in fibrotic regions in the late phase of cardiac fibrosis. (6) In cardiomyopathy patients, versican isoforms V0 and V1, as well as the DPEAAE fragment, are upregulated and accumulated in the fibrotic regions.

Pressure overload induced by aortic banding is a well-established experimental mouse model enabling examination of cardiac hypertrophy with fibrosis and development of cardiac dysfunction leading to heart failure [19]. In that model, we found that expression of versican was upregulated immediately after initiation of aortic banding, i.e. after day 1, and that it preceded the expression of collagen types I and III, indicating that versican is important for the transition from provisional matrix to fibrotic matrix [27]. Previous studies by our group have reported elevated levels of versican in pressure overload [7], and by others in heart failure [12], which are disease conditions associated with cardiac fibrosis. To the best of our knowledge, our study is the first to assess the temporal and spatial profile of versican expression and its isoforms, as well as its cleavage product during the development of cardiac fibrosis.

Our data demonstrated that V1 is the main isoform expressed in the heart during fibrosis development. During extracellular matrix remodeling, an increase in the V1 isoform has been associated with proliferation [28] and activation of fibroblasts [29]. Moreover, the V1 isoform induces recruitment of inflammatory cells [30], which are involved in the inflammation-fibrosis axis in the heart [31], [32], [33]. Hence, an early induction of the V1 isoform following pressure overload may participate in the inflammatory response. Thus, alterations in the V1 isoform levels might regulate cardiac fibrosis.

Using *in situ* hybridization, we found that cardiac fibroblasts, cardiomyocytes, and non-cardiomyocytes are producers of versican in the initial phase following aortic banding. However, towards the later phase of ECM remodeling, versican synthesis was restricted to cardiac fibroblasts. These results indicate that cardiac fibroblasts are the main cellular origin of versican [12] during cardiac fibrosis. The contribution of other cells [12] in the initial phase could be related to the inflammatory response following aortic banding [18], [19], [20], [34]. We also found that increased mechanical stretch of fibroblasts and stimulation with TGF-β, a central pro-fibrotic growth factor [35], increased versican levels in cardiac cells. An increase in versican expression by TGF-β in arterial smooth muscle cells [36], [37], and by TNF-α in resting endothelial cells [38] has been reported. In a cardiac disease condition, either a combination of mechanical strain and cytokine stimulation, or the initial change in mechanical strain that occurs in cardiac diseases may induce versican synthesis.

Elevated levels of versican were observed mainly in the perivascular area 3 days after aortic banding. Perivascular fibrosis is frequently observed following aortic banding [39]. However, we did not detect fibrosis around the vessels at this early stage (day 3) following aortic banding. Instead, perivascular fibrosis evolved after the increase in versican production from day 14 to 56 in the aortic banded hearts. Previous studies have reported that versican precedes collagen expression in pulmonary fibrosis [40], and that it modulates collagen fibrillogenesis by direct binding [41]. Thus, in perivascular fibrosis, an early deposition of versican around the vessels may be important for the propagation of cardiac fibrosis from the vessels into the myocardium, leading to interstitial fibrosis [42].

Cleavage of versican by ADAMTS (a disintegrin and metalloproteinase with thrombospondin motifs) enzymes -1, -4, and -5 is a central process during cardiac remodeling [12], [43], [7]. Our findings indicate that the ADAMTS4 enzyme contributes importantly to increased levels of DPEAAE fragment after aortic banding and are in accordance with our previously published study [14]. The relevance of versican cleavage in embryonic development, as seen in interdigital web regression [44], limb [45] and cardiac development [46] is well documented. However, information about the spatial distribution and temporal profile of versican cleavage in cardiac fibrosis is not known. We found that the cleaved fragment of the V1 isoform, DPEAAE, was significantly increased in the later stages of fibrosis development in the pressure overloaded hearts. Moreover, the DPEAAE fragment was accumulated in replacement, interstitial and perivascular fibrosis in the heart. An elevated level of the DPEAAE fragment in ischemic injury has been reported, but not the spatial distribution of the fragment [12]. The cleavage of versican might have a functional role [25], where accumulation of the fragment induces immune cell infiltration [47]. One previous study has reported that increased cleavage of versican worsened colitis in the gut, compared to animals with less versican cleavage [24]. Thus, our data suggest an important role for the versican cleavage fragment in the later phases of matrix remodeling induced by pressure overload.

Importantly, the use of myocardial samples from patients with heart disease strengthens the translational value of our study. As in mice with aortic banding, we found that V0 and V1 isoforms of versican were upregulated in patients with cardiomyopathies. Moreover, versican and its fragment, DPEAAE, were deposited in fibrotic regions with replacement, interstitial and perivascular fibrosis in the myocardium. An increased deposition of chondroitin sulfate chains, where versican is the largest chondroitin sulfate proteoglycan, in the extracellular matrix of end-stage heart failure patients has been reported [8]. Moreover, targeting versican through chondroitin sulfate chains [8] or its cleavage has improved cardiac function in a heart failure model [7], [14]. Our study showing the dynamic profile of versican and DPEAAE in cardiac fibrosis development may contribute to further development of heart failure therapies.

Taken together, versican, mainly the V1 isoform, is expressed early after initiation of pressure overload by collagen producing fibroblasts, suggesting that versican might be important for early cardiac remodeling process. Moreover, versican cleavage into the DPEAAE fragment progresses toward the later phases of cardiac fibrosis development following pressure overload. In patients with cardiomyopathies, elevated expression and accumulation of versican and the DPEAAE fragment were observed in fibrotic regions of the myocardium. Thus, combining our findings from pressure overload in mice and myocardial samples from cardiomyopathy patients indicates that versican and the DPEAAE fragment are involved in different phases of cardiac fibrosis development.

## Materials and methods

### O-ring aortic banding (AB)

Male C57BL/6NCrl mice (9-weeks old, Charles River Laboratories, Sulzfeld, Germany) were used for AB and sham (Control) operations as described previously [19]. Left ventricles of the hearts were excised and snap frozen for protein and RNA measurement. Cryoblocks of the biventricular base of the hearts were prepared using OCT (Chemi-Teknik AS, Norway, #21860), and stored at −80°C for histology.

All animals were treated in accordance with the National Institute of Health Guide for the Care and Use of Laboratory Animals (2011), and the procedures were approved by the Norwegian Food Safety Authority (FOTS ID: 20793). ARRIVE guidelines were followed to report animal research. For terminal experiments and prior to tissue harvest, mice were anesthetized with 5% isoflurane.

### Echocardiography

An experienced researcher blinded to the experimental procedure captured echocardiographic images of left ventricular dimension of the heart using a VEVO 3100 (VisualSonics, Toronto, Canada) [48] in freely breathing mice anaesthetized with 1.75% isoflurane. Left ventricular wall thickness, fractional shortening (FS), left atrial diameter (LAD) and left ventricular ejection fraction (LVEF) were assessed. LVEF and FS were calculated from the M-mode by Vevo LAB software (v.5.6.1, VisualSonics).

### Immunoblotting

The extracellular matrix (ECM) fraction was extracted from snap frozen left ventricular tissue for detection of versican. The tissue was homogenized in 1% SDS buffer with 31.5 mM Tris-HCl (pH 6.8) containing a protease inhibitor cocktail (cOmplete™, Mini, EDTA-free tablets, Roche Diagnostics, Merck Sigma, Germany, #11836170001) and PhosSTOP (Roche Diagnostics, Merck Sigma, Germany, #4906837001). To isolate versican from the ECM fraction, the insoluble pellet from the SDS fraction was incubated overnight with 5 M guanidine lysis buffer with 50 mM sodium acetate and 2.5 mM EDTA (pH 5.8) at −20°C. Then, the lysate was treated overnight with chondroitinase ABC enzyme (AMS Biotechnology Limited, United Kingdom, #AMS.E1028-02, 0.5 U/ml) in deglycosylation buffer (50 mM Tris HCl and 60 mM sodium acetate) at 37°C to remove glycosaminoglycan chains. An equal concentration of the lysate (37 μg) was loaded onto a 4–15% gradient gel (Bio-Rad, #5671085) under reducing conditions, and separated by SDS-PAGE. Proteins were transferred to polyvinylidene difluoride membranes (Bio-Rad, #1704157) using the Trans-Blot Turbo system (Bio-Rad). The membranes were blocked with 5% non-fat milk for 1 h, and incubated with primary antibodies: Rabbit anti-mouse versican (GAG-β) antibody (Merck Sigma, Germany, #AB1033, 1:1000), and rabbit anti-mouse/human versican V0, V1 neo-polyclonal antibody (Invitrogen, #PA1-1748A, 1:1000) at 4°C overnight. The membranes were washed three times with TBS-T (Tris-buffered saline with 1% Tween-20) (Bio-Rad, #1610781) for 10 min each. Then, they were incubated with HRP conjugated donkey-anti-rabbit secondary antibody (Cytiva, MA, #NA934, 1:3000) for 1 h, followed by TBS-T wash for 10 min (three times). The membranes were developed using the Supersignal^TM^ West Pico PLUS chemiluminescent substrate (ThermoFisher Scientific, #34580), and visualized by the Azure 600 western blot imaging system (Azure Biosystems, CA). Protein bands were normalized using the total amount of protein per lane, which was stained using Revert™ 700 Total Protein Stain (Li-COR Biotechnology, Germany, #926–11021). Pooled ECM fractions to detect versican protein levels and 1% SDS fractions to detect DPEAAE fragment from mouse cardiac tissue obtained 8 to 10 weeks after AB operations were used as positive controls.

### Immunofluorescence

Immunofluorescent staining was performed as previously [49] with modifications as described below. Cryosections of 7 μm thickness from the mid-ventricular plane were fixed in pre-cooled methanol (70%) acetone (30%) solution at −20°C for 10 min. The sections were blocked in Protein Block (Serum-Free, Agilent Dako, #X090930-2) for 10 min and incubated overnight with primary antibodies rabbit anti-mouse versican (GAG-β) antibody (Merck Sigma, Germany, #AB1033, 1:1000), rat anti-human versican antibody (R&D systems, MN, #MAB3054, 1:100), and rabbit anti-mouse/human versican V0, V1 neo-epitope antibody (Invitrogen, #PA1-1748A, 1:1000) at 4°C. Next day, sections were washed twice with 0.05% Tween-20 in PBS, followed by a PBS wash for 10 min each at room temperature. Then, the sections were incubated with goat anti-rabbit Alexa Flour™ Plus 647 (Invitrogen, #A32733, 1:400), goat anti-rat Alexa Fluor™ Plus 647 (Invitrogen, #A48265, 1:400) or goat anti-rabbit Alexa Fluor™ Plus 488 (Invitrogen, #A-11034, 1:200) secondary antibodies for 1 h at room temperature. Alexa Flour® 488 conjugated (Invitrogen, #W11261, 1:200) or Alexa Flour® 555 conjugated (Invitrogen, #W32464, 1:200) wheat germ agglutinin (WGA) was applied to the sections for 10 min, followed by a PBS wash. Then, the sections were stained with DAPI (Merck Sigma, Germany, #MBD0015, 1:1000) for 10 min followed by a PBS wash. Vector TrueVIEW Autofluorescence Quenching Kit (Vector Laboratories, CA, #SP-8400-15) was used to reduce background signal in accordance with the manufacturer’s protocol. The sections were mounted using Mowiol mounting medium (Merck Sigma, Germany, 81381). Z-stack images (n = 8 images per sample in humans, n = 4–6 vessels per animal for perivascular fibrosis in mouse) were captured at 63x oil objective using LSM 800 Airyscan microscope (Zeiss), and quantified using CellProfiler software (v.4.2.1, https://www.cellprofiler.org). We designed an automated pipeline to measure the positive area of versican, DPEAAE, and WGA staining. The measured areas of versican and DPEAAE were normalized to WGA, and relative value was calculated with respective controls. For quantification of versican in perivascular fibrosis in mouse, the positive stained area was normalized to the perimeter of each vessel. Negative antibody staining for mouse and human cardiac tissue is shown in Fig. S3D-U.

For hyaluronan staining, cryosections were fixed in ethanol (70%), glacial acetic acid (5%), and formaldehyde (4%) solution at room temperature for 10 min [50]. As a negative control for hyaluronan staining, the sections were treated with hyaluronidase from *Streptomyces hyalurolyticus* (Merck Sigma, Germany, #H1136, 50U/ml) in 75 mM sodium chloride, 20 mM sodium acetate buffer, pH 6.0 at 37°C for overnight (Fig. S5E-G). All samples were incubated with sodium acetate buffer alone at 37°C for overnight. Then, the sections were incubated with ReadyProbes™ Streptavidin/Biotin Blocking Solution (Invitrogen, #R37628) for 20 min each, followed by Protein Block (Serum-Free, Agilent Dako, #X090930-2) for 10 min, and incubated overnight with biotinylated hyaluronic acid binding protein (HABP, Merck Sigma, Germany, #385911) at 4°C. Next day, the sections were incubated with Alexa Fluor™ 647 conjugated streptavidin (Invitrogen, #S32357, 1:200) for 1 h at room temperature. The sections were washed, stained with DAPI, and mounted using Mowiol mounting medium as described above. Samples incubated with HABP only or Alexa Fluor™ 647 conjugated streptavidin only were used as negative controls for hyaluronan staining (Fig. S5A-D).

### Histological fibrosis quantification

Cryosections of mouse tissue (7 μm thickness) were stained with Masson’s trichrome (Polysciences, Germany, #25088-1) in accordance with the manufacturer’s protocol. The images of whole hearts were captured at 20x objective on Axioscan Z1 (Carl Zeiss, Germany). Quantification of Masson’s trichrome images have been described previously [51]. For perivascular fibrosis quantification, positive area of collagen staining around the vessel was normalized to the perimeter of each vessel using QuPath (v.0.3.0, https://qupath.github.io/).

### RNA isolation and gene expression analysis

Total RNA was isolated from the tissue and cell cultures using RNeasy mini kit (Qiagen Nordic, Norway, #74106) as described in the manufacturer’s protocol. The quality and concentration of RNA were analysed by a 2100 Bioanalyzer Instrument (Agilent Technologies, CA, #G2938C) and Multiscansky (ThermoFisher scientific), respectively. cDNA was synthesized using the iScript cDNA Synthesis Kit (Bio-Rad Laboratories, CA, #1708891). The primers used for human and mouse versican and for the isoforms V0, V1, V2, V3 have been published [52], [53]. The primers used for human and mouse collagen I and III were: *COL1A2* (Hs01028956_m1), *COL3A1* (Hs00943809_m1), *Col1a2* (Mm00483888_m1) and *Col3a1* (Mm00802331_m1). The primers used for mouse ADAMTS enzymes were: *Adamts4* (Mm00556068_m1), *Adamts1* (Mm01344169_m1) and *Adamts5* (Mm00478620_m1). Generation of droplets for digital PCR and reading of the amplified droplets were done using QX200 AutoDG Droplet Digital PCR system (Bio-Rad). Data were analyzed using QuantaSoft™ Software (Bio-Rad Laboratories, v.1.7.4). Gene expression was normalized to glyceraldehyde 3-phosphate dehydrogenase (*GAPDH*, Hs02786624_g1) for humans, and the absolute quantity of mRNA expression (copies/μl) was used for the mouse studies. For quantitative real time-PCR of cell cultures, we used TaqMan gene expression assays for *VCAN* (Hs00171642_m1), *ACTA2* (Hs00426835_g1), and the 60S ribosomal protein L32 (*RPL32*, Hs00851655_g1, as housekeeping gene).

### Single molecular fluorescence *in situ* hybridization (smFISH)

smFISH was performed on mouse cardiac cryosections (7 μm thickness) with custom made HuluFISH probes for versican (Atto 488-*Vcan*) and collagen 1 (Atto 647-*Col1a1*) as described in the manufacturer’s protocol (PixelBiotech, HuluFISH Kit, Germany). FISH probes targeting green fluorescent protein (GFP) mRNA labelled with Atto 488 or Atto 647 were used as negative controls. Deviations from the protocol are mentioned below. The cryosections were fixed in 4% formaldehyde (ThermoFisher Scientific, #28906) in PBS for 10 min, followed by PBS wash. The sections were additionally permeabilized with pre-cooled methanol (70%) acetone (30%) solution at −20°C for 10 min, and rinsed briefly with PBS. After HuluWash and staining with the probes, the sections were washed thrice for 1 h to remove unbound probes. Sections were mounted with ProLong™ Glass Antifade Mountant with NucBlue™ Stain (ThermoFisher Scientific, #P36983). Images were captured at 63x using an oil objective on LSM 800 Airyscan microscope (Zeiss).

### Human cardiac cells and cytokine treatment

Fetal human cardiac fibroblasts (CFs) (Cell Applications, CA, #306-05F) and primary human cardiomyocytes (CMs) (PromoCell, Germany, #C-12810) were used for *in vitro* experiments. CFs were cultured in cardiac fibroblast growth medium (Cell Applications, CA, #316-500) containing 1% penicillin and streptomycin (Merck Sigma, Germany, #P0781) at 37°C. For serum free conditions, fibroblast basal medium (Cell Applications, CA, #115-500) was used. Prior to cytokine treatment, CFs were cultured for 24 h at a concentration of 2.5 × 10^5^ cells/well on 2 kPa silicone plates (ExCellness Biotech SA, Switzerland, #01.035.002.00) to reduce myofibroblast activation. Then, cells were serum starved for 3 h, and treated with cytokines at a concentration of 10 ng/ml for 21 h at 37°C. The cells were rinsed with PBS, and harvested using RNeasy lysis buffer for RNA extraction. CMs were cultured in myocyte growth medium (PromoCell, Germany, #C-22070) with 10% fetal bovine serum (Biosera, Nuaille, France, #FB-1001), and 1% penicillin and streptomycin at 37°C. Myocyte basal medium (PromoCell, Germany, #C-22270) was used for serum free conditions. CMs were cultured on plastic plates at the same cell concentration and conditions as CFs.

The cytokines used for the experiment were recombinant human tumor necrosis factor-α (TNF-α, #210-TA-005), recombinant human interleukin (IL)-1β (#201-LB-005), recombinant human IL-4 (#204-IL-010), recombinant human IL-13 (#213-ILB-005), recombinant human IL-17A (#7955-IL025), recombinant human IL-10 (#217-IL-005), recombinant human CCL2 (#279-MC-010), recombinant human IL-18 (#9124-IL), and recombinant human transforming growth factor beta (TGF-β1, Merck Sigma, Germany, #1141227200). We used 0.1% BSA as control for the experiment. The cytokines were purchased from R&D systems (MN).

### Mechanical stretch of fibroblasts

For mechanical stretching of CFs, we used 6-well Bioflex® culture plates (Dunn Labortechnik, Asbach, Germany, #BF-3001U) coated with fibronectin bovine plasma (Merck Sigma, Darmstadt, Germany, #F1141) at a concentration of 2 μg/ml for 24 h. The cells were cultured at a concentration of 3.5 × 10^5^ cells/well for 24 h at 37°C in cardiac fibroblast growth medium (Cell Applications, CA, #316-500). Prior to the cell stretch experiment, the cell culture medium was changed to fibroblast basal medium (Cell Applications, CA, #115-500). A biaxial strain of 10% at a frequency of 1 Hz was applied for 24 h using the FX-6000T™ Tension system (Flexcell® International Corporation, NC). The cells were rinsed with PBS and harvested using RNeasy lysis buffer for RNA extraction. Non-stretched cells cultured at the same conditions were used as controls.

### Myocardial samples from cardiomyopathy patients

We used left ventricular myocardial samples from patients with hypertrophic obstructive cardiomyopathy (HOCM, n = 13, collected during septal myectomy), dilated cardiomyopathy (DCM, n = 11, collected from the free wall after explantation), and donors not used for transplantation as control hearts (Control, n = 4) to examine versican and its isoforms. Human cardiac tissue was processed in a similar manner as mouse tissue for RNA isolation and immunostaining, as described above. The clinical characteristics of the hypertrophic obstructive cardiomyopathy patients have been published previously [54], [55], and information about patients with dilated cardiomyopathy is presented in Supplementary Table 1. The use of myocardial samples was approved by the Regional Committee for Medical and Health Research Ethics (REK S-02295, 07482a, S-05172), and is in accordance with the Declaration of Helsinki.

### Statistical analysis

Data presented represent mean ± standard deviation (SD). Statistical tests (GraphPad Prism 9, CA) used were one or two-way analysis of variance (ANOVA) with Dunnett’s or Bonferroni’s multiple comparisons tests, unpaired Student’s *t*-test, or Welch’s *t*-test if there was significant difference in variance by Fisher’s exact test as indicated in the figure legend. Shapiro-Wilk test was used to check the distribution of the data and parametric tests were used accordingly. Pearson correlation coefficient was used for correlation analysis. For patient data, outliers (DCM = 1 sample, Control = 1 sample) were identified and removed using Grubb’s test (alpha = 0.05) provided by Graphpad (outlier calculator). P values < 0.05 were considered statistically significant.

## Sources of funding

This study was funded by K.G. Jebsen Center for Cardiac Research, South-Eastern Norway Regional Health Authority, Fondsstiftelsen Oslo University Hospital and Norwegian Health Association. Andreas Romaine has received funding from the European Union’s Horizon 2020 research and innovation programme under the Marie Skłodowska-Curie grant agreement No 801133.

## CRediT authorship contribution statement

**Athiramol Sasi:** Conceptualization, Methodology, Formal analysis, Validation, Investigation, Project administration, Visualization, Resources, Writing – original draft. **Andreas Romaine:** Conceptualization, Methodology, Validation, Investigation, Resources, Writing – review & editing. **Pugazendhi Murugan Erusappan:** Conceptualization, Methodology, Formal analysis, Validation, Investigation, Resources, Writing – review & editing. **Arne Olav Melleby:** Methodology, Writing – review & editing. **Almira Hasic:** Methodology, Formal analysis, Validation, Investigation, Resources. **Christen Peder Dahl:** Investigation, Resources, Writing – review & editing. **Kaspar Broch:** Investigation, Resources, Writing – review & editing. **Vibeke Marie Almaas:** Investigation, Resources, Writing – review & editing. **Rosa Doñate Puertas:** Methodology, Investigation, Resources, Writing – review & editing. **H. Llewelyn Roderick:** Methodology, Investigation, Resources, Writing – review & editing. **Ida Gjervold Lunde:** Investigation, Resources, Writing – review & editing. **Ivar Sjaastad:** Investigation, Resources, Funding acquisition, Methodology, Writing – review & editing. **Maria Vistnes:** Conceptualization, Supervision, Writing – review & editing. **Geir Christensen:** Conceptualization, Methodology, Investigation, Project administration, Visualization, Funding acquisition, Supervision, Writing – review & editing.

## Declaration of Competing Interest

The authors declare the following financial interests/personal relationships which may be considered as potential competing interests: The University of Oslo has filed a patent application (WO2015004209A1) for the use of ADAMTS4 inhibition in heart failure and cardiac remodeling that has been granted in the United States (US10744155B2) and is pending in Europe (EP3756667A1). The authors (Vistnes M, Christensen G, Lunde IG, Sjaastad I) of this manuscript are named as inventors in this patent. The authors declare that they have no other conflicts of interest with the contents of this article.

## Data Availability

Data will be made available on request.
