# Supplementary material for: Temporal expression and spatial distribution of the proteoglycan versican during cardiac fibrosis development
Source: Matrix Biol Plus. 2023 Nov 10;19-20:100135. doi: 10.1016/j.mbplus.2023.100135 (PMC10709089; doi:10.1016/j.mbplus.2023.100135)
Supplement: Supplementary data 1 [file mmc1.pdf]

Supplementary figures and table

Temporal expression and spatial distribution of the proteoglycan versican during cardiac fibrosis development

Athiramol Sasi [a,b *], Andreas Romaine [a,b], Pugazendhi Murugan Erusappan [a], Arne Olav Melleby [a], Almira Hasic [a], Christen Peder Dahl [c], Kaspar Broch [b,d], Vibeke Marie Almaas [d], Rosa Doñate Puertas [e], H. Llewelyn Roderick [b,e], Ida Gjervold Lunde [a,g,h], Ivar Sjaastad [a,b], Maria Vistnes [a,f], Geir Christensen [a,b]

a - *Institute for Experimental Medical Research,* *Oslo University Hospital and University of Oslo, Oslo, Norway*
b - *K.G. Jebsen Center for Cardiac Research,* *University of Oslo, Oslo, Norway*
c - *Research Institute of Internal Medicine,* *Oslo University Hospital, Oslo, Norway*
d - *Department of Cardiology,* *Oslo University Hospital Rikshospitalet, Oslo, Norway*
e - *Experimental Cardiology,* *Department of Cardiovascular Sciences, KU Leuven, Leuven, Belgium*
f - *Department of Cardiology,* *Oslo University Hospital Ullevål, Oslo, Norway*
g - *Center for Clinical Heart Research,* *Department of Cardiology, Oslo University Hospital Ullevål, Oslo, Norway*
h - *K.G. Jebsen Center for Cardiac Biomarkers,* *Institute for Clinical Medicine, Campus Ahus, University of Oslo, Oslo, Norway*

Correspondence to Athiramol Sasi: Institute for Experimental Medical Research, Oslo University Hospital and University of Oslo, Oslo, Norway, *athiramol.sasi@medisin.uio.no* (Athiramol Sasi)

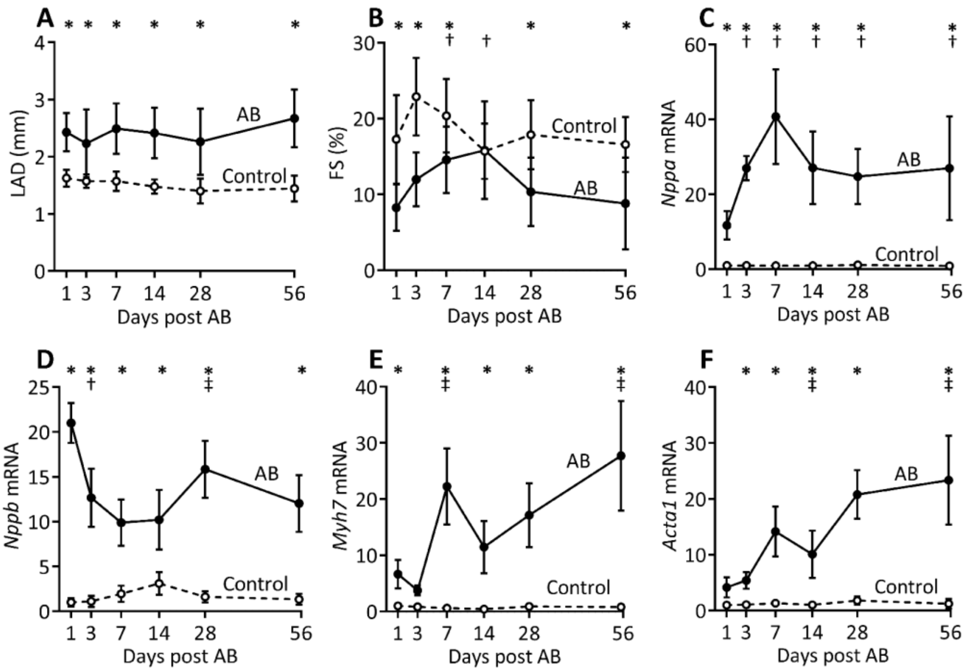

Fig. S1. Echocardiographic measurements and markers of cardiac remodeling in pressure overload. (A) Left atrial diameter (LAD) and (B) fractional shortening (FS) in aortic banding (AB, n = 16-18 at day 1 to 28, n = 8 at day 56), and sham (Control, n = 18-20 at day 1 to 28, n = 10 at day 56) hearts. Relative mRNA expression of (C) atrial natriuretic peptide (*ANP*), (D) B-type natriuretic peptide (*BNP*), (E) myosin heavy chain 7 (*Myh7*) and (F) actin alpha skeletal muscle 1 (*Acta1*) from the left ventricle of mouse hearts at day 1, 3, 7, 14, 28 and 56 after AB (n = 8-11) and from Control (n = 9-10) hearts. The absolute quantity of gene expression (copies/µl measured by ddPCR) relative to Control at day 1 is presented. Data represent mean ± SD. Repeated measures two-way ANOVA with Bonferroni's multiple comparisons test was used for statistical analysis. P values <0.05 were considered statistically significant. *P<0.05 Control vs. AB, †P<0.05 AB vs. day 1 post AB, ‡P<0.05 AB vs. day 3 post AB.

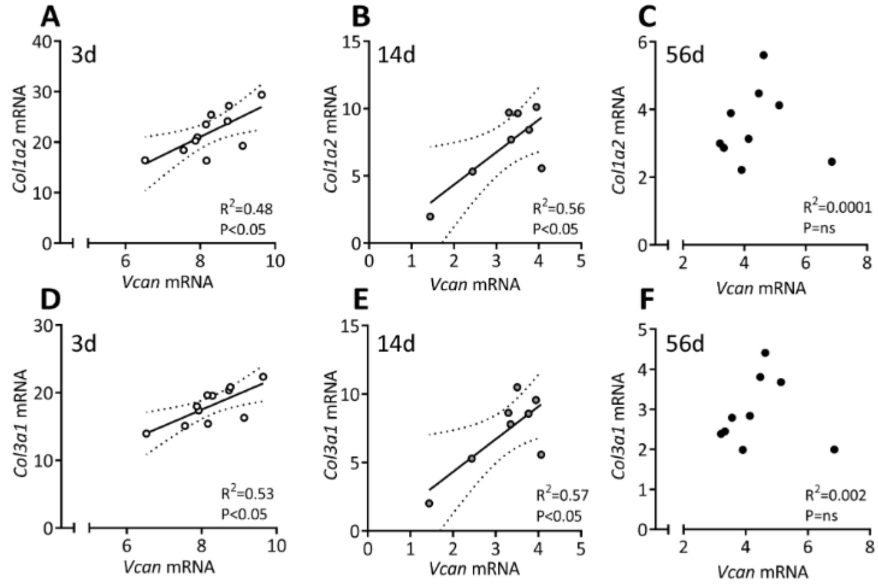

Fig. S2. Versican (*Vcan*) and collagen correlate positively in the early phase of fibrosis development. (A-C) Correlation (R^2) between relative mRNA expression of versican (*Vcan*) and collagen I alpha 2 (*Col1a2*), and (D-F) collagen III alpha 1 (*Col3a1*) at day 3, 14 and 56 after aortic banding. Pearson correlation coefficient was used for statistical analysis. P values <0.05 were considered statistically significant. ns: not significant.

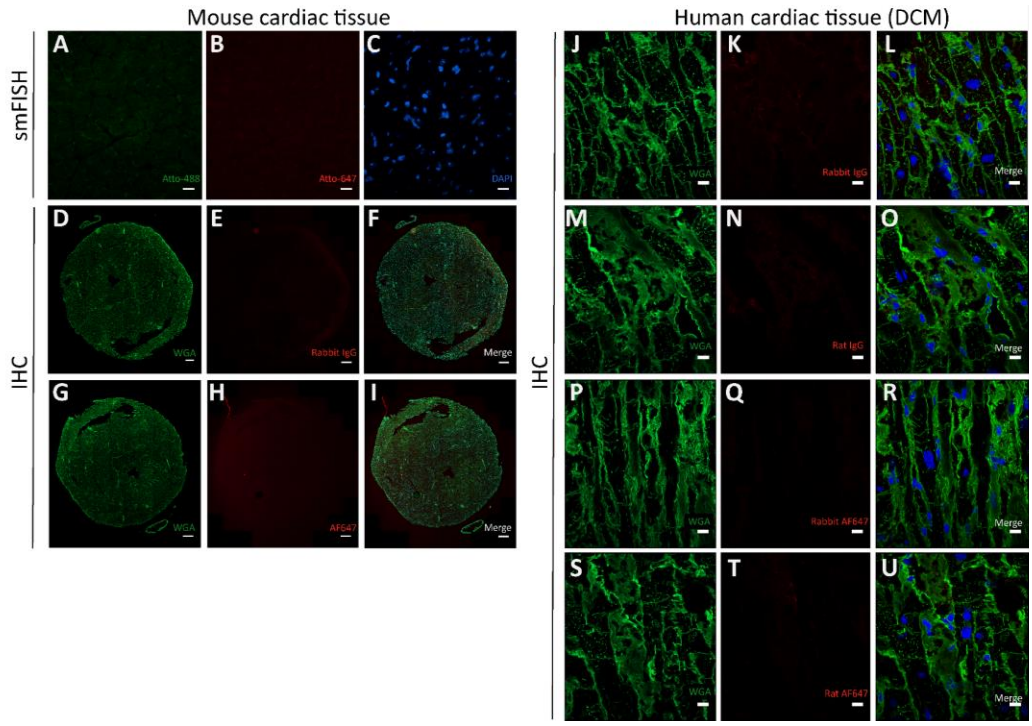

Fig. S3. Antibody control staining for smFISH and immunofluorescence staining. (A-C) Representative confocal image for green fluorescent protein (GFP) mRNA labelled with (A) Atto-488 (green) as a control for versican probe, (B) Atto-647 (red) as a control for collagen 1 probe, and (C) DAPI for nuclei (blue) were used for

smFISH experiments. Scale bar = 15 µm. (D-F) Rabbit IgG control (red) was used as a negative control for versican (#AB1033) and DPEAAE (#PA1-1748a) antibody staining in mouse cardiac tissue. (G-I) Mouse cardiac tissue stained only with goat anti-rabbit Alexa Flour^{TM} Plus 647 (AF647, red) was used as secondary antibody control. Scale bar = 240 µm. (J-L) Rabbit IgG control (red) was used as a negative control for DPEAAE (#PA1-1748a) antibody staining, and (M-O) Rat IgG control (red) was used as a negative control for versican (#MAB3054) antibody staining in human tissue. (P-R) Goat anti-rabbit Alexa Flour^{TM} Plus 647 (Rabbit AF647, red), and (S-U) goat anti-rat Alexa Flour^{TM} Plus 647 (Rat AF647, red) were used as secondary antibody controls in human tissue. Wheat germ agglutinin (WGA, green) stain for the cardiomyocyte sarcolemma and extracellular matrix and DAPI stain for the nuclei (blue). Scale bar = 15 µm.

Table S1. Dilated cardiomyopathy patient characteristics

Patient characteristics (n=11)	
Female gender	3 (27)
Age (years)	43.1±15.7
BMI	23±2.3
Current smoker	1 (9)
Duration of heart failure (years)	6.7±5.9
NYHA functional class IV	4 (27)
**Heart failure therapy**	
CRT	3 (27)
ACE-I or ARB	10 (90)
Betablocker	7 (63)
MRA	5 (45)
**Clinical chemistry**	
Hemoglobin (g/dL)	12.8±1.3
Creatinine (µmol/L)	101.1±36.1
NT-proBNP (pg/mL)	483.4±661.5
**Echocardiography**	
IVSd (cm)	0.7±0.19
LVPWd (cm)	0.7±0.2
LVIDd (cm)	7.2±0.9
LVIDs (cm)	6.8±0.9
CO (L/min)	3±0.9
EF (%)	18.5±2.9
E/A	2.7±1.1
Right atrium (mmHg)	5.8±3
PA mean (mmHg)	23.2±10.5
PCWP	13.7±8.3
RV CO	4.5±0.9

Values are presented as mean ± standard deviation, and presented as percent in parentheses. BMI: body mass index, NYHA: New York Heart Association, CRT: cardiac resynchronisation therapy, ACE-I: Angiotensin-converting enzyme-inhibitors, ARB: angiotensin II receptor blockers, MRA: mineralocorticoid receptor antagonist, NT-proBNP: N-terminal pro-brain natriuretic peptide, IVSd: interventricular septum thickness in diastole, LVPWd: left ventricle posterior wall thickness in diastole, LVIDd: left ventricle internal diameter in diastole, LVIDs: left ventricle internal diameter in systole, CO: cardiac output, EF: ejection fraction, E/A: E-wave to A-wave ratio, PA: pulmonary artery, PCWP: pulmonary capillary wedge pressure, RV: right ventricle.

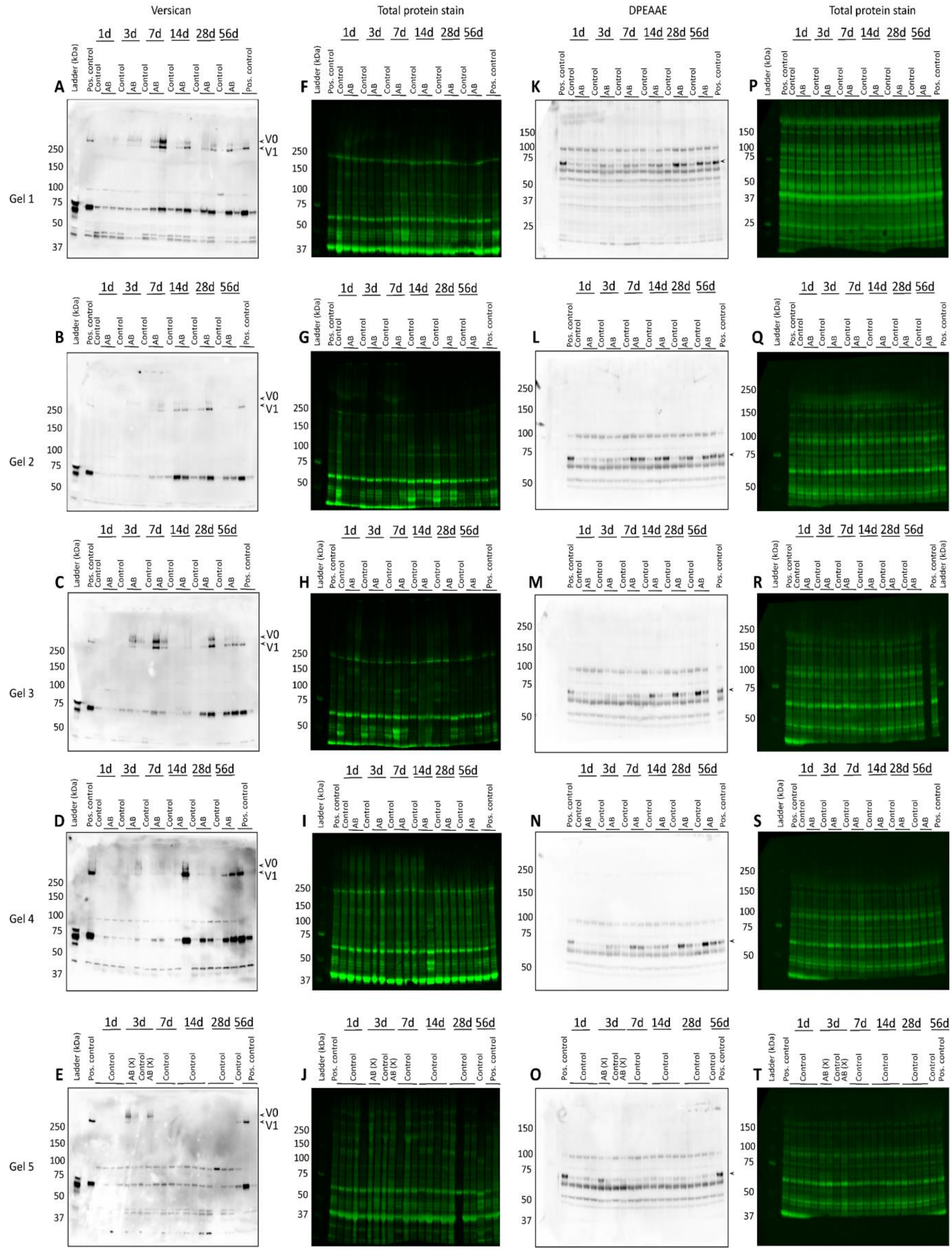

Versican
Total protein stain
DPEAAE
Total protein stain
1d 3d 7d 14d 28d 56d
Ladder (kDa)
Pos. control
Control
AB
AB (X)
A
B
C
D
E
F
G
H
I
J
K
L
M
N
O
P
Q
R
S
T
Gel 1
Gel 2
Gel 3
Gel 4
Gel 5
V0
V1
250
150
100
75
50
37
25

Fig. S4. Immunoblot for versican, DPEAAE, and total protein stain of mouse cardiac tissue after sham operation (Control) and aortic banding (AB). Complete western blot images showing the protein expression of (A-E) versican (black arrows show V0 and V1 isoforms), (F-J) total protein stain of versican immunoblots, (K-O) DPEAAE (black arrow), and (P-T) total protein stain of DPEAAE immunoblots from the left ventricles of mice at day 1, 3, 7, 14, 28 and 56 after aortic banding (AB, n = 8) or sham operations (Control, n = 5-8). The samples were treated with chondroitinase ABC enzyme (0.5U/ml) to remove glycosaminoglycan chains, except the sample on the right side of the image. Pooled ECM fractions to detect versican protein levels and 1% SDS fractions to detect DPEAAE fragment from mouse cardiac tissue obtained 8 to 10 weeks after AB operations were used as positive controls. For quantification of versican, AB samples (n = 8) were relative to the average of day 1 AB. Control samples (sham operation) displayed very low or no amount of versican. For quantification of the DPEAAE fragment, AB samples (n = 8) were related to the average of AB at day 1, and Control samples (n = 5-8) were related to Control values at day 1. The samples marked with "x" (E, J, O, T) were not included in the quantification of versican and DPEAAE western blot due to the absence of appropriate control (day 1 AB).

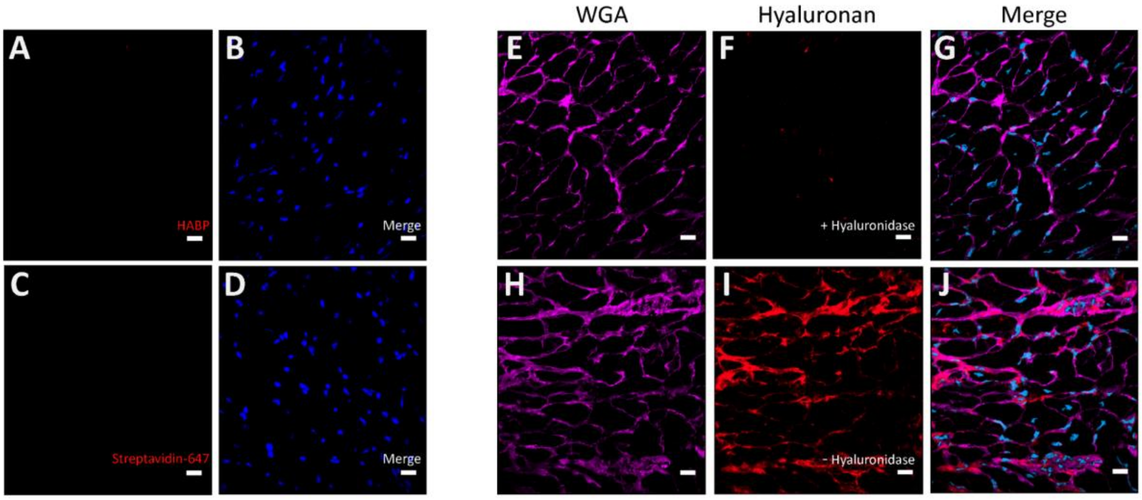

Fig. S5. Negative controls for hyaluronan staining. (A-D) Representative confocal images for (A-B) biotinylated hyaluronic acid binding protein only (HABP), (C-D) Alexa Fluor™ 647 conjugated streptavidin only (Streptavidin-647), (E-G) cryosections treated with hyaluronidase, and (H-J) without hyaluronidase were used as negative controls for hyaluronan staining. Wheat germ agglutinin (WGA, magenta) stain for the cardiomyocyte sarcolemma and extracellular matrix, hyaluronan (red), and DAPI stain for the nuclei (blue). Scale bar = 15 µm.
